# Supplementary material for: Single Cell Sequencing Reveals Mechanisms of Persistent Truncus Arteriosus Formation after PDGFRα and PDGFRβ Double Knockout in Cardiac Neural Crest Cells
Source: Genes (Basel). 2022 Sep 23;13(10):1708. doi: 10.3390/genes13101708 (PMC9601305; doi:10.3390/genes13101708)
Supplement: Supplementary file 1 [file genes-13-01708-s001.zip › Supplement Figures S1-17 and Table S1.pdf]

Figure S1 Proportion of surviving embryo genotypes at E17.5 and P0

Genotype identification of these survival embryos showed that all *Pax3*$^{cre/+}$*;PDGFRα*$^{fl/+}$*;PDGFRβ*$^{fl/fl}$ genotype embryos could not survive to birth, other genotypes were inherited according to the Mendelian law.

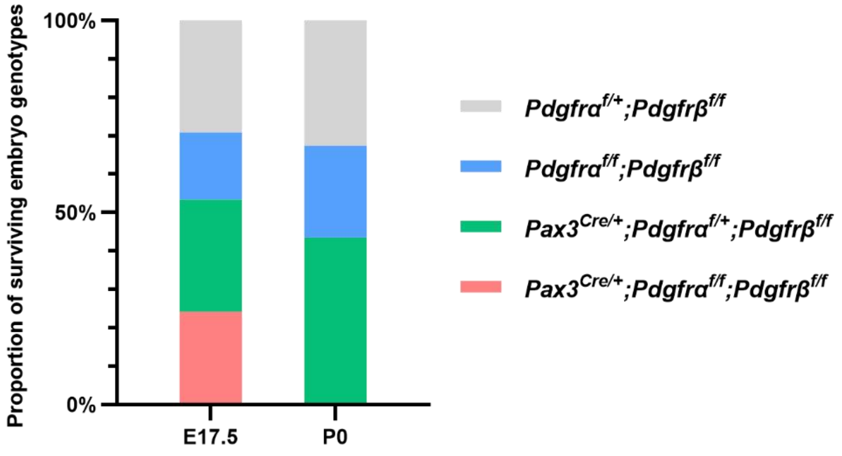

Figure S2 Phenotype of *PDGFRα* single knockout group at E17.5

Coronal-section pathological slides with HE staining showed that there were a range of heart phenotypes including normal, PTA, and DORV after *PDGFRα* knockout in CNCCs. PTA, persistent truncus arteriosus; DORV, double outlet of right ventricle. Scale bar, 300 µm.

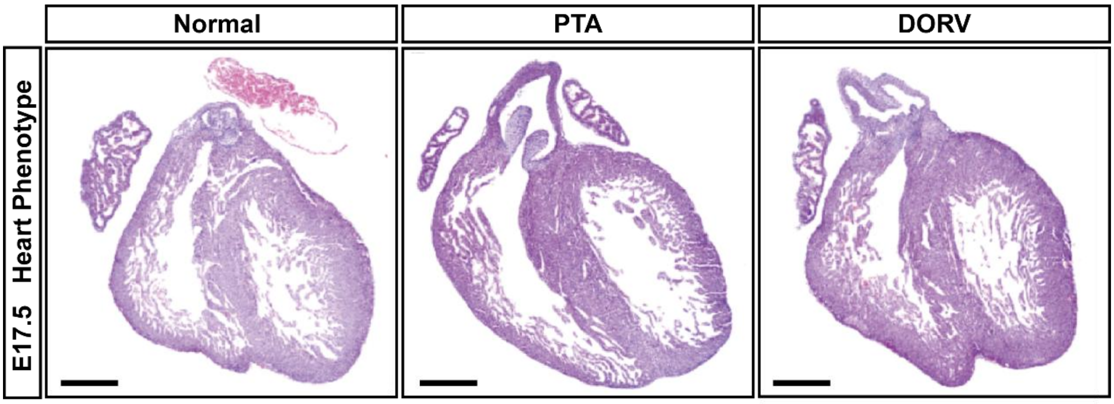

Figure S3 Phenotype of *PDGFRβ* single knockout group at E17.5

Coronal-section pathological slides with HE staining showed that there were a range of heart phenotypes including normal and VSD after *PDGFRβ* knockout in CNCCs. VSD, ventricular septal defect. Scale bar, 300 μm.

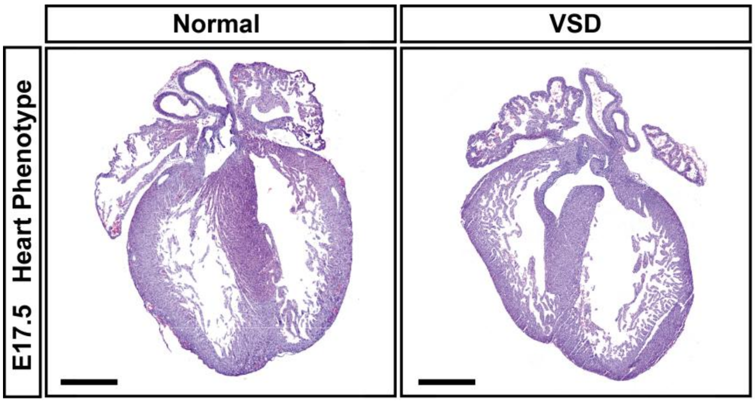

Figure S4 Phenotypic of *PDGFRα* heterozygous knockdown group at E17.5

Coronal-section pathological slides with HE staining showed that conotruncal phenotypes of $Pax3^{Cre/+}$;$PDGFR\alpha^{fl/+}$;$PDGFR\beta^{+/+}$ embryos were normal. Scale bar, 300 μm.

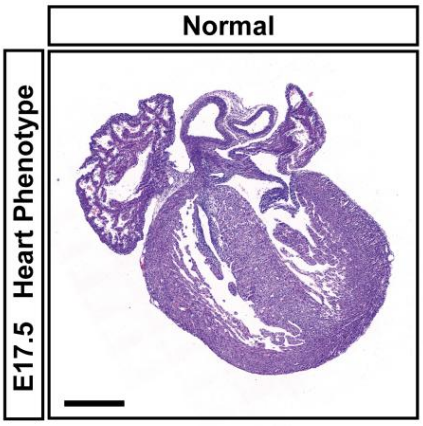

Figure S5 Immunolabeling for CNCC-RFP, and DAPI staining on transverse sections across the OFT at three distinct distal-proximal levels in E12.5 embryos with the indicated genotype.

The result showed that there was no significant difference between CON (*Pax3$^{cre/+}$;PDGFRα$^{fl/+}$;PDGFRβ$^{fl/+}$*) and DKO (*Pax3$^{cre/+}$;PDGFRα$^{fl/fl}$;PDGFRβ$^{fl/fl}$*) group in the number of *RFP*$^{+}$ CNCCs per section. CON, control; DKO, double knockout; OFT: outflow tract. Scale bar, 50 μm.

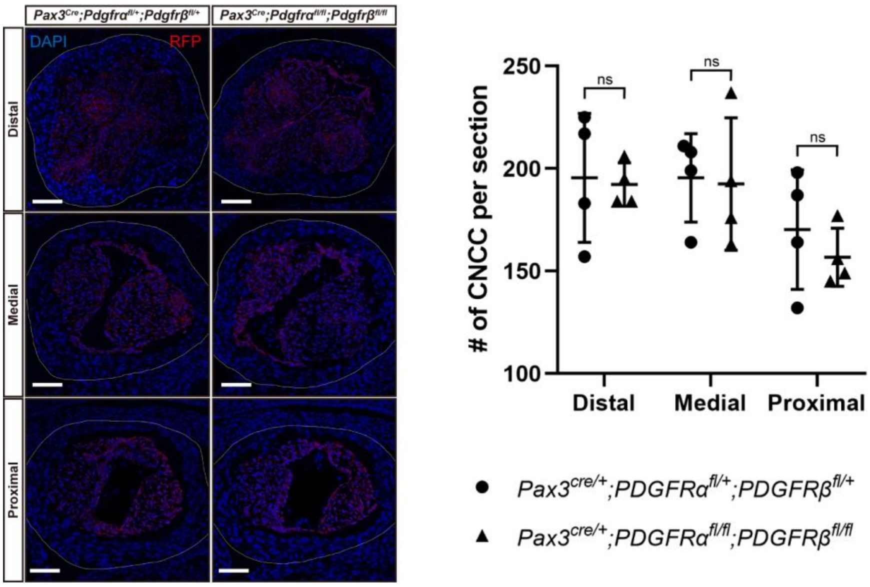

Figure S6 Transverse sections of E12.5 OFT stained for Ki67 between DKO and control group.

The result showed that there was no significant difference between CON (*Pax3$^{cre/+}$;PDGFRα$^{fl/+}$;PDGFRβ$^{fl/+}$*) and DKO (*Pax3$^{cre/+}$;PDGFRα$^{fl/fl}$;PDGFRβ$^{fl/fl}$*) group in the number of Ki67^{+} CNCCs per section. CON, control; DKO, double knockout; OFT: outflow tract. Scale bar, 50 μm.

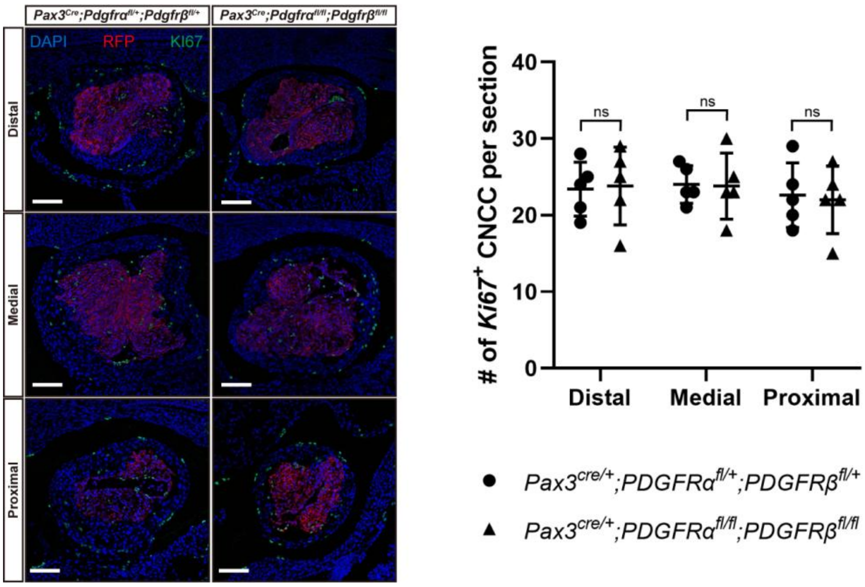

Figure S7 Transverse sections of E12.5 OFT stained for pH3 between DKO and control group.

The result showed that there was no significant difference between CON (*Pax3*$^{cre/+}$*;PDGFRα*$^{fl/+}$*;PDGFRβ*$^{fl/+}$) and DKO (*Pax3*$^{cre/+}$*;PDGFRα*$^{fl/fl}$*;PDGFRβ*$^{fl/fl}$) group in the number of pH3^{+} CNCCs per section (white arrow). CON, control; DKO, double knockout; OFT: outflow tract; pH3: phospho-histone H3. Scale bar, 50 µm.

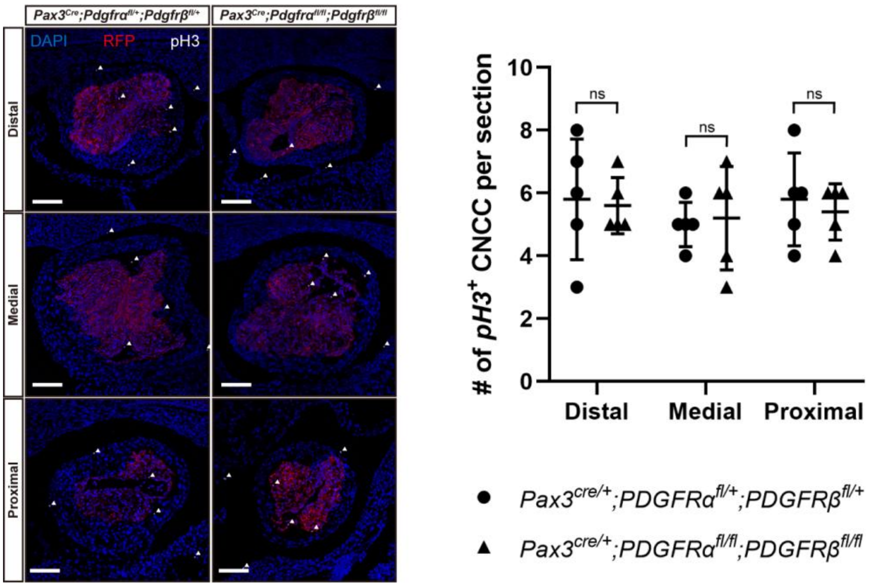

Figure S8 Transverse sections of E12.5 OFT stained for TUNEL between DKO and control group.

The result showed that there was no significant difference between CON (*Pax3*$^{cre/+}$*;PDGFRα*$^{fl/+}$*;PDGFRβ*$^{fl/+}$) and DKO (*Pax3*$^{cre/+}$*;PDGFRα*$^{fl/fl}$*;PDGFRβ*$^{fl/fl}$) group in the number of TUNEL^{+} CNCCs per section (green arrow). CON, control; DKO, double knockout; OFT: outflow tract. TUNEL: terminal deoxynucleotidyl transferase-mediated dUTP-biotin nick end labeling. Scale bar, 50 µm.

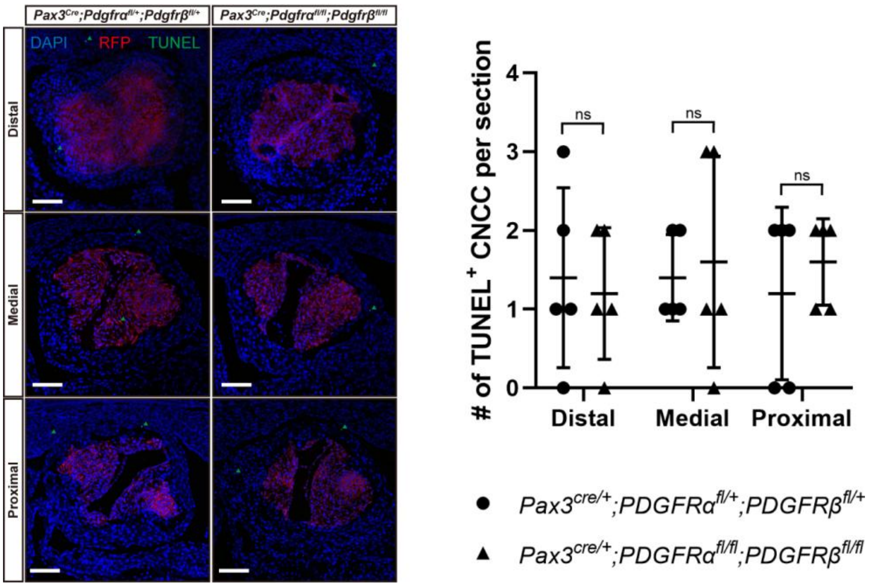

Figure S9 Quality filtering of single-cell RNA sequencing.

Cells that were of low quality or represented doublets were filtering out by exclusion criteria: 1) greater than 25000 and fewer than 600 genes; 2) greater than 4000 and fewer than 350 UMI counts; 3) greater than 20% mitochondrial gene proportion; and 4) greater than or equal to 40% ribosomal gene proportion in Seurat.

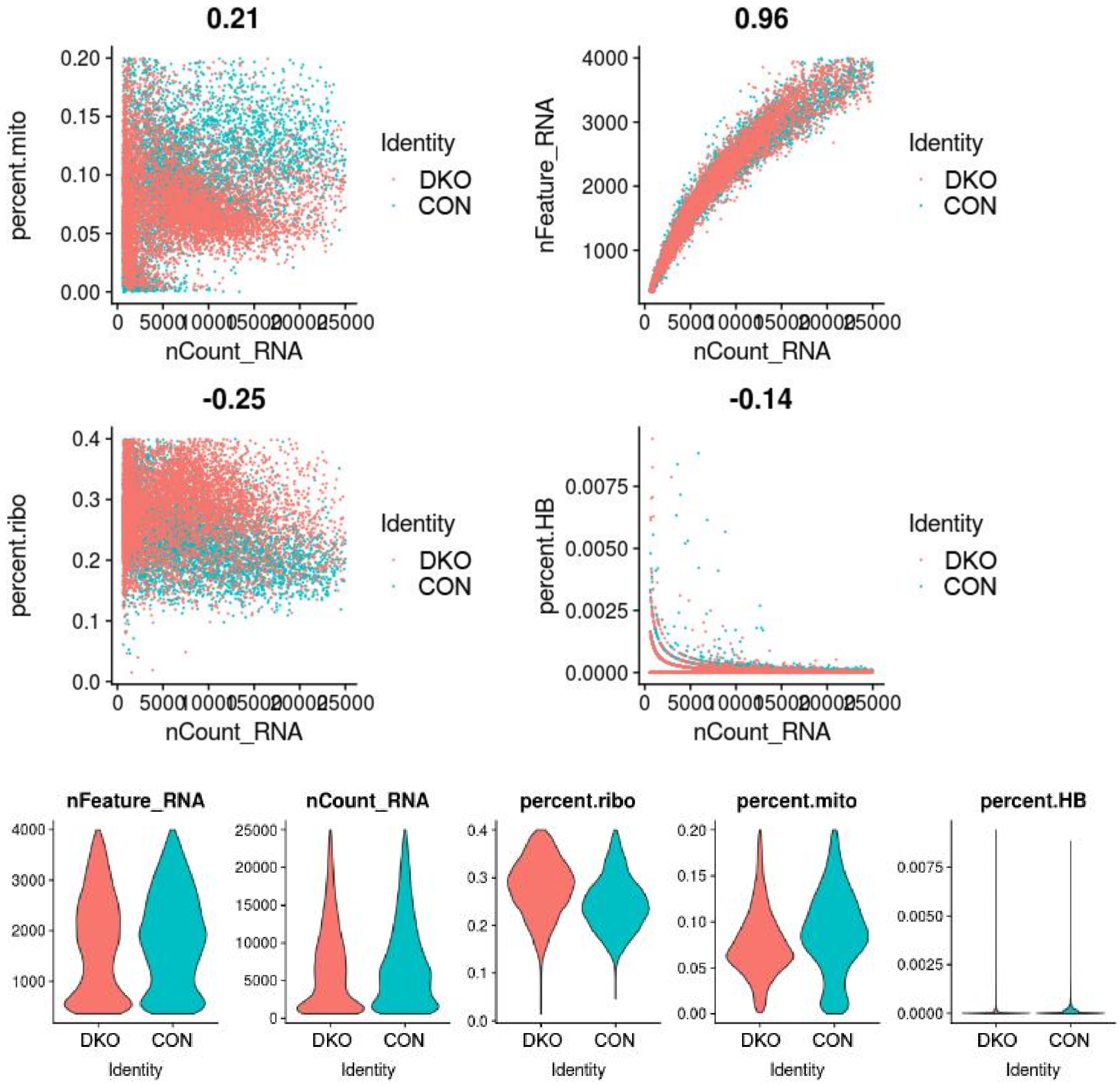

Figure S10 UMAP plots of all captured OFT cells colored by groups.

UMAP plots of all captured OFT cells colored by groups showed that cells from CON and DKO groups were mixed evenly, and there was no obvious batch effect. CON, control; DKO, double knockout; UMAP: uniform manifold approximation and projection; OFT: outflow tract.

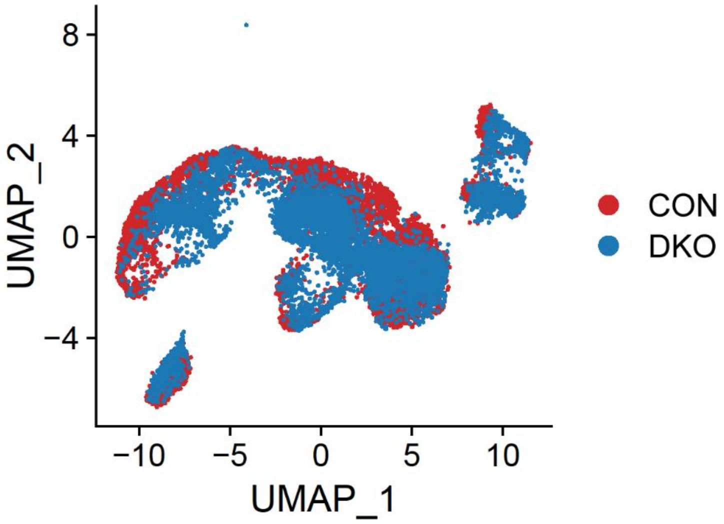

Figure S11 Cell cluster distribution of DKO and control group on UMAP plots.

Cell cluster distribution of DKO and control group on UMAP plots showed that there were significant differences in the number of cells from CON and DKO groups. CON, control; DKO, double knockout; MS, mesenchymal cell; CM, cardiomyocyte; VSMC, vascular smooth muscle cell; EP, epicardial cell; EC: endothelial cell; UMAP: uniform manifold approximation and projection.

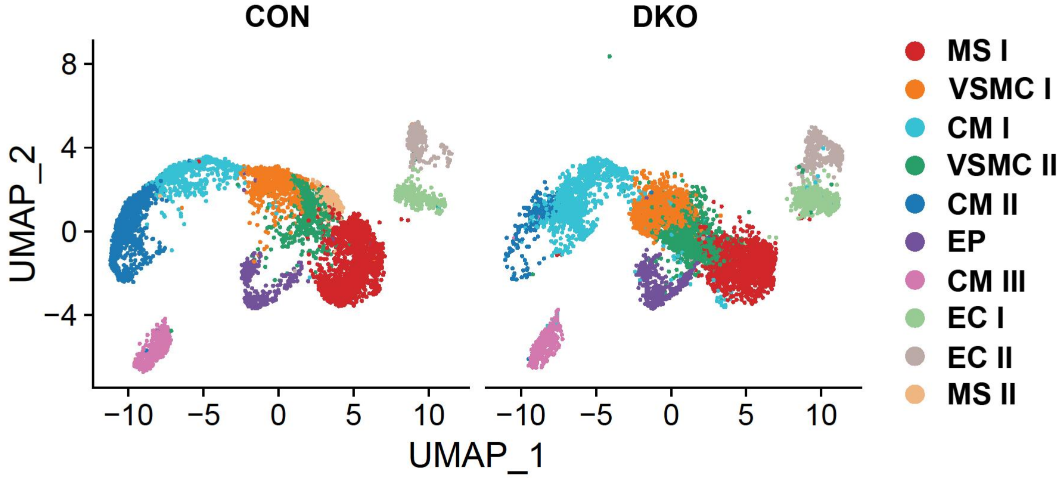

Figure S12 Direction and rate of cellular state changes inferred by RNA velocity analysis. The result revealed that there were two major cell differentiation trends among cell clusters in OFT: CM to VSMC (CM I to VSMC I via CM II) and MS to VSMC (MS I to VSMC II via MS II). CON, control; DKO, double knockout; MS, mesenchymal cell; CM, cardiomyocyte; VSMC, vascular smooth muscle cell; EP, epicardial cell; EC: endothelial cell; OFT, outflow tract.

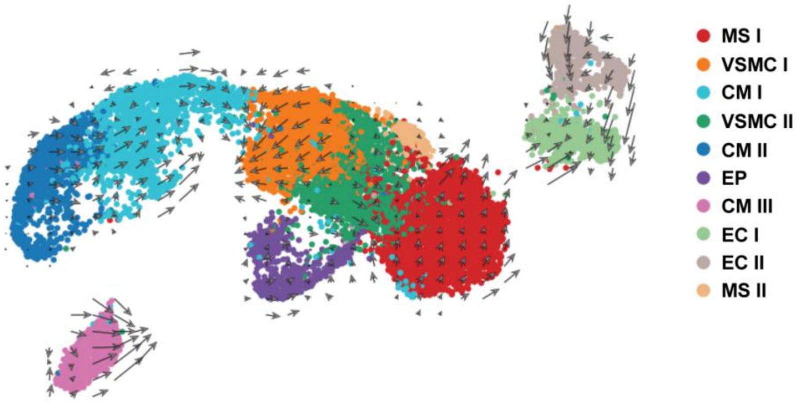

Figure S13 The function of OFT cell clusters in PDGF signaling pathway indicated by CellChat.

CellChat analysis results revealed that in the PDGF signaling pathway network, CM I cluster was the main sender, MS I was the receiver and MS II was the mediator.

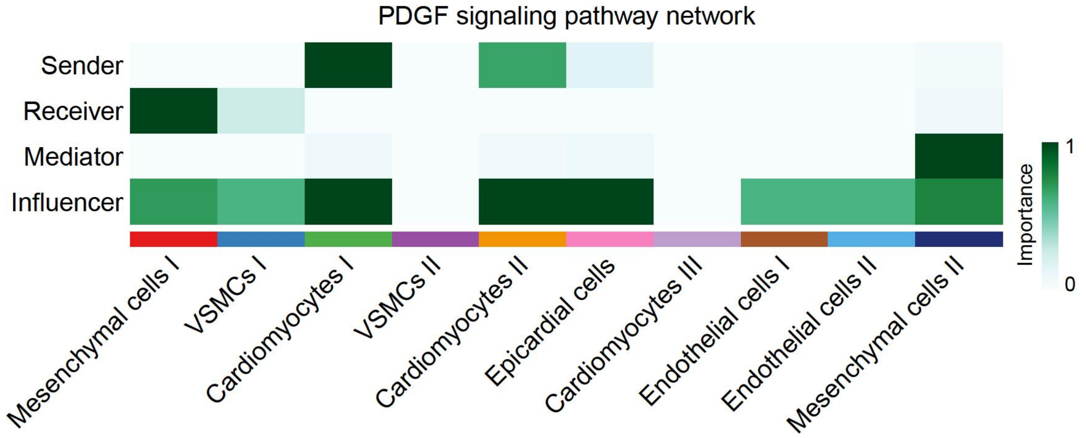

Figure S14 *In vivo* immunofluorescence verification experiment with Penk and CNCC-RFP.

The result showed that all the *Penk*$^{+}$ MS II cells had red fluorescence used for CNCC lineage-tracing in the control group and were absent in the DKO group. Scale bar, 50 µm.

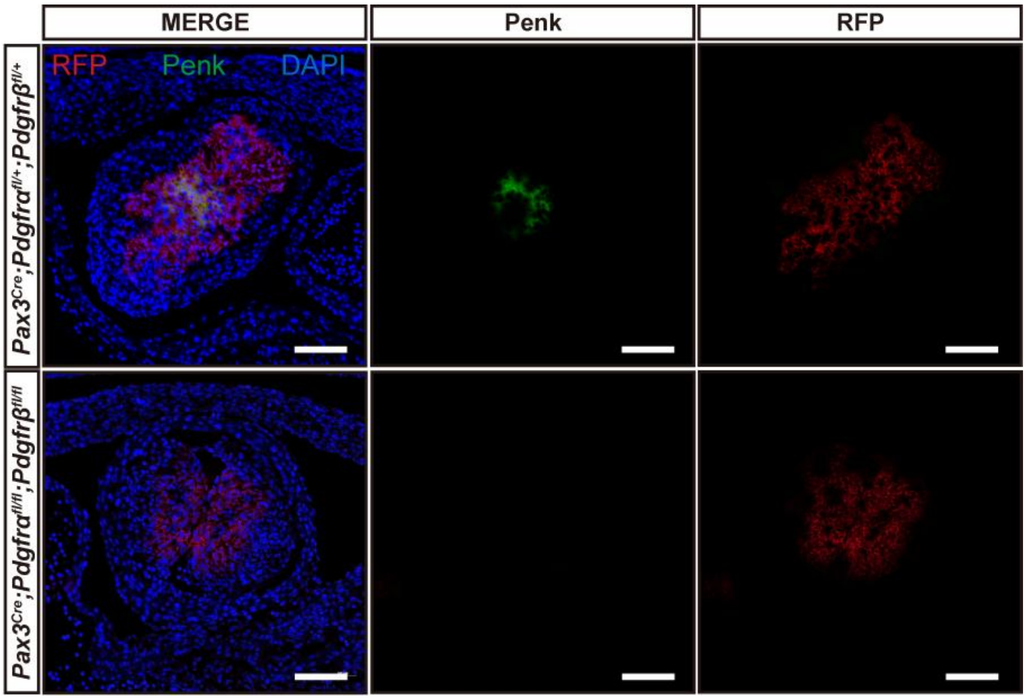

Figure S15 Co-analysis with single cell data from *Liu et al. 2019.*

The result revealed that *Penk*+ MS II cells were derived from CNCCs according to reference data, and absent in DKO group. REF: reference data; CON, control; DKO, double knockout.

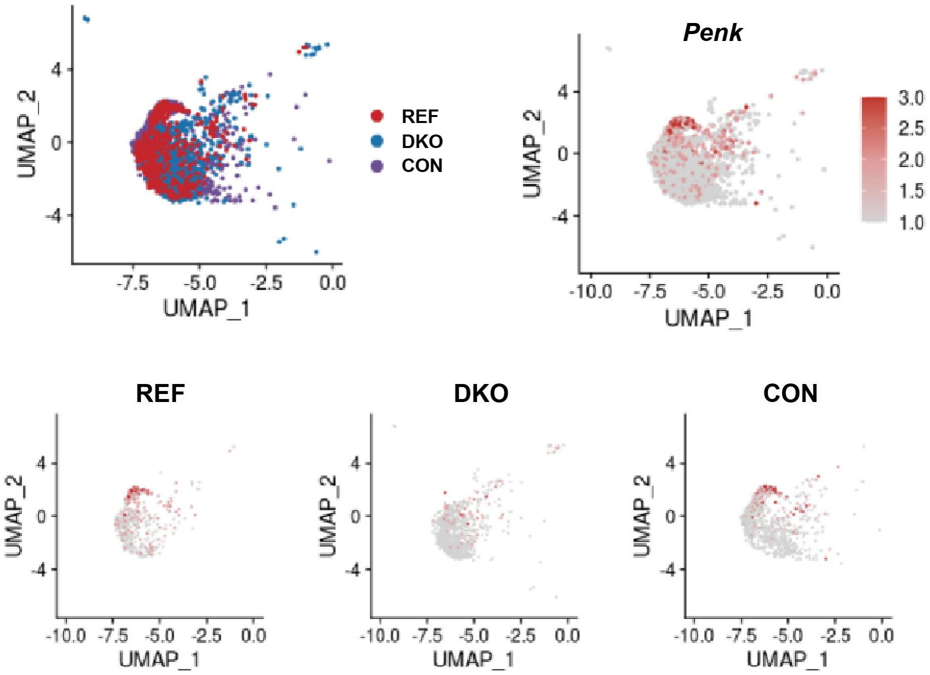

Figure S16 Expression of marker genes across VSMC clusters as visualized on UMAP plots.

The result revealed that VSMC I specifically expressed *Col1a2* and *Fbn1* and VSMC II highly expressed *Tmsb10* and *Tmsb4x*. VSMC, vascular smooth muscle cell; UMAP: uniform manifold approximation and projection.

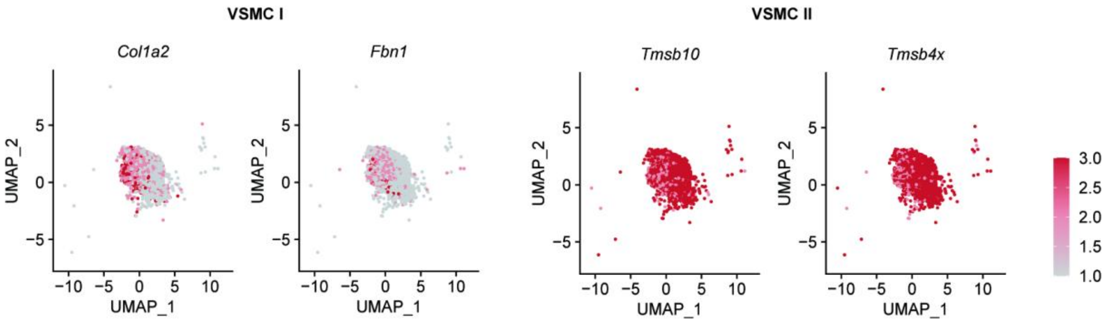

Figure S17 Comparation of ligand-receptor interactions among MS, CM and VSMC clusters between the DKO and control group by CellChat.

M, mesenchymal cell; C, cardiomyocyte; V, vascular smooth muscle cell; CON, control; DKO, double knockout.

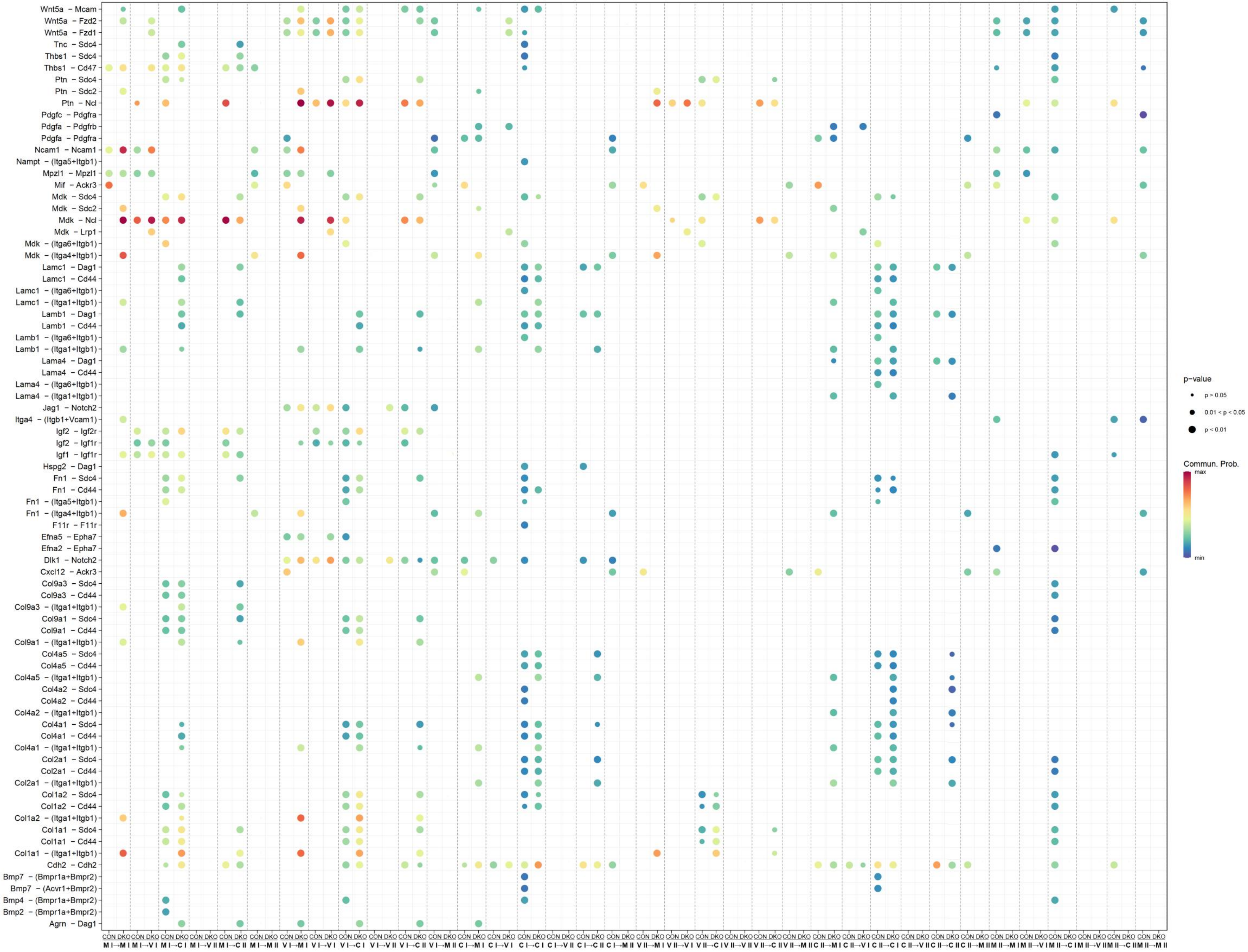
Wnt5a - Mcam
Wnt5a - Fzd2
Wnt5a - Fzd1
Tnc - Sdc4
Thbs1 - Sdc4
Thbs1 - Cd47
Ptn - Sdc4
Ptn - Sdc2
Ptn - Ncl
Pdgfc - Pdgfra
Pdgfa - Pdgfrb
Pdgfa - Pdgfra
Ncam1 - Ncam1
Nampt - (Itga5+Itgb1)
Mpzl1 - Mpzl1
Mif - Ackr3
Mdk - Sdc4
Mdk - Sdc2
Mdk - Ncl
Mdk - Lrp1
Mdk - (Itga6+Itgb1)
Mdk - (Itga4+Itgb1)
Lamc1 - Dag1
Lamc1 - Cd44
Lamc1 - (Itga6+Itgb1)
Lamc1 - (Itga1+Itgb1)
Lamb1 - Dag1
Lamb1 - Cd44
Lamb1 - (Itga6+Itgb1)
Lamb1 - (Itga1+Itgb1)
Lama4 - Dag1
Lama4 - Cd44
Lama4 - (Itga6+Itgb1)
Lama4 - (Itga1+Itgb1)
Jag1 - Notch2
Itga4 - (Itgb1+Vcam1)
Igf2 - Igf2r
Igf2 - Igf1r
Igf1 - Igf1r
Hspg2 - Dag1
Fn1 - Sdc4
Fn1 - Cd44
Fn1 - (Itga5+Itgb1)
Fn1 - (Itga4+Itgb1)
F11r - F11r
Efna5 - Epha7
Efna2 - Epha7
Dlk1 - Notch2
Cxcl12 - Ackr3
Col9a3 - Sdc4
Col9a3 - Cd44
Col9a3 - (Itga1+Itgb1)
Col9a1 - Sdc4
Col9a1 - Cd44
Col9a1 - (Itga1+Itgb1)
Col4a5 - Sdc4
Col4a5 - Cd44
Col4a5 - (Itga1+Itgb1)
Col4a2 - Sdc4
Col4a2 - Cd44
Col4a2 - (Itga1+Itgb1)
Col4a1 - Sdc4
Col4a1 - Cd44
Col4a1 - (Itga1+Itgb1)
Col2a1 - Sdc4
Col2a1 - Cd44
Col2a1 - (Itga1+Itgb1)
Col1a2 - Sdc4
Col1a2 - Cd44
Col1a2 - (Itga1+Itgb1)
Col1a1 - Sdc4
Col1a1 - Cd44
Col1a1 - (Itga1+Itgb1)
Cdh2 - Cdh2
Bmp7 - (Bmpr1a+Bmpr2)
Bmp7 - (Acvr1+Bmpr2)
Bmp4 - (Bmpr1a+Bmpr2)
Bmp2 - (Bmpr1a+Bmpr2)
Agrn - Dag1
CON DKO
M I→M I
M I→V I
M I→C I
M I→V II
M I→C II
M I→M II
V I→M I
V I→V I
V I→C I
V I→V II
V I→C II
V I→M II
C I→M I
C I→V I
C I→C I
C I→V II
C I→C II
C I→M II
V II→M I
V II→V I
V II→C I
V II→V II
V II→C II
V II→M II
C II→M I
C II→V I
C II→C I
C II→V II
C II→C II
C II→M II
M II→M I
M II→V I
M II→C I
M II→V II
M II→C II
M II→M II
p-value
p > 0.05
0.01 < p < 0.05
p < 0.01
Commun. Prob.
max
min

Table S1 Phenotypic statistics of *PDGFRα* knockout, *PDGFRβ* knockout, and double knockout group at E17.5.

	Normal	Isolated VSD	Isolated DORV	DORV+VSD	PTA+VSD	Total
Pax3$^{Cre/+}$;Pdgfrα$^{f/f}$	7 (8%)	7 (8%)	8 (9%)	41 (44%)	29 (31%)	92
Pax3$^{Cre/+}$;Pdgfrβ$^{f/f}$	35 (95%)	2 (5%)	0	0	0	37
Pax3$^{Cre/+}$;Pdgfrα$^{f/f}$;Pdgfrβ$^{f/f}$	0	0	0	0	48 (100%)	48

PTA, persistent truncus arteriosus; DORV, double outlet of right ventricle; VSD, ventricular septal defect.
